# 血友病患者出血急诊管理中国指南（2024年版）

**DOI:** 10.3760/cma.j.cn121090-20240809-00296

**Published:** 2024-10

**Authors:** 中华医学会血液学分会血栓与止血学组 中国血友病协作组

## Abstract

血友病是一种X染色体连锁的隐性遗传性出血性疾病。出血是血友病患者最常见的急症，也是血友病患者致死、致残、影响生活质量的主要因素。快速识别和规范处置对于改善患者预后具有重要意义。急诊科是血友病患者出血的主要首诊科室，血友病出血急诊处置流程复杂，常常需要多学科联合诊疗和综合管理。为了规范国内从事血友病诊疗的各相关科室的诊疗行为，中华医学会血液学分会血栓与止血学组和中国血友病协作组共同制定了本指南。

## Abstract (en)

Hemophilia is a X-linked recessive hemorrhagic disease. Bleeding is the most common complication of hemophilia, and it is also the main cause leading to death and disability or reducing the quality of life of hemophilia patients. Rapid identification and standardized management of the bleeding events is of great significance to improve the prognosis of hemophilia patients. Emergency department is the frontline department for hemophilia patients with bleeding. The emergent management process of hemophilia hemorrhage is complex and often needs multidisciplinary team cooperation. To increase the awareness of the related professionals who may involve in the management of bleeding events of hemophilia patients, in collaboration with the Thrombosis and Hemostasis Group, Chinese Society of Hematology, Chinese Medical Association, Hemophilia Treatment Center Collaborative Network of China issued the Chinese guidelines for emergency management of bleeding in hemophilia patients.

血友病是一种罕见的X染色体连锁隐性遗传性出血性疾病，分为血友病A（占80％～85％）和血友病B（占15％～20％），分别由凝血因子Ⅷ（FⅧ）和凝血因子Ⅸ（FⅨ）缺乏引起[1]。出血是血友病患者最常见的急症，也是血友病患者致死、致残、影响生活质量的主要因素[2]。血友病患者的临床出血表现多样，可以为自发性出血，也可以为手术或创伤后出血不止[2]。急诊科是血友病急症的主要首诊科室，美国四家急救中心的数据显示血友病患者年急诊就诊人数占全年总急诊人数的0.02％[3]。血友病出血急诊处置流程复杂，需要多学科联合诊疗和综合管理[4]。中华医学会血液学分会血栓与止血学组和中国血友病协作组组织国内相关专家历时1年余讨论后制订本指南，旨在为血友病的急诊规范化诊疗提供指引。

一、血友病出血急诊管理原则及流程

出血急诊管理基本原则：怀疑出血即治疗；尽快开始凝血因子替代治疗；尽快识别危及生命的大出血或关键部位出血；有针对性地采集病史和选择辅助检查；兼顾出血相关并发症的处置；重视多学科协作诊疗（MDT），实施个体化综合管理（出血急诊管理流程见图1）。

**图1:**
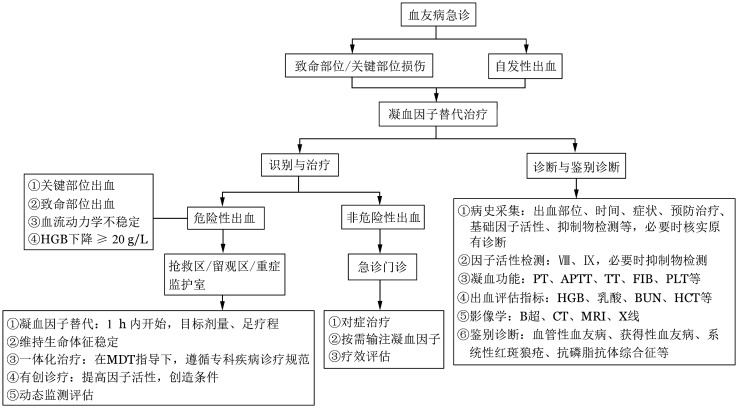
血友病患者出血急诊管理流程 **注** APTT：活化部分凝血活酶时间；PT：凝血酶原时间；TT：凝血酶时间；FIB：纤维蛋白原；MDT：多学科诊疗；MRI：磁共振成像

1. 尽快开始凝血因子替代治疗：血友病患者出血的严重程度与凝血因子水平密切相关[2],[5]。重型血友病患者一旦发生出血，必须立即、足量予以凝血因子替代治疗，尽快控制出血，不必等待辅助检查结果而延误替代治疗开始时间。轻型及中间型血友病发生创伤性出血或面临手术等有创操作时也需要凝血因子替代治疗（具体方案见表1、表2）。建议于出血发生后1 h内开始凝血因子输注[2]。

**表1:** 获取凝血因子不受限时的替代治疗方案[1]

出血类型	血友病A		血友病B	
	预期水平（%）	疗程（d）	预期水平（%）	疗程（d）
关节	40~60	1~2（若反应不充分可延长）	40~60	1~2（若反应不充分可延长）
浅表层肌肉（无神经血管损伤）	40~60	2~3（若反应不充分可延长）	40~60	2~3（若反应不充分可延长）
髂腰肌和深层肌（或伴神经血管损伤）				
初始	80~100	1~2	60~80	1~2
维持	30~60	3~5	30~60	3~5
中枢神经系统/头部				
初始	80~100	1~7	60~80	1~7
维持	50	8~21	30	8~21
颈、咽喉部				
起始	80~100	1~7	60~80	1~7
维持	50	8~14	30	8~14
胃肠道				
起始	80~100	7~14	60~80	7~14
维持	50		30	
肾脏	50	3~5	40	3~5
深部裂伤	50	5~7	40	5~7
手术（大）				
术前	80~100		60~80	
术后	60~80	1~3	40~60	1~3
	40~60	4~6	30~50	4~6
	30~50	7~14	20~40	7~14
手术（小）				
术前	50~80		40~50	
术后	30~80	1~5	10~40	1~5

**表2:** 获取凝血因子受限时的替代治疗方案[1]

出血类型	血友病A		血友病B	
	预期水平（%）	疗程（d）	预期水平（%）	疗程（d）
关节	10~20	1~2（若反应不充分可延长）	10~20	1~2（若反应不充分可延长）
浅表层肌肉（无神经血管损伤）	10~20	2~3（若反应不充分可延长）	10~20	2~3（若反应不充分可延长）
髂腰肌和深层肌（或伴神经血管损伤）				
初始	20~40	1~2	15~30	1~2
维持	10~20	3~5	10~20	3~5
中枢神经系统/头部				
初始	50~80	1~3	50~80	1~3
维持	30~50	4~7	30~50	4~7
	20~40	7~14	20~40	7~14
咽喉和颈部				
起始	30~50	1~3	30~50	1~3
维持	10~20	4~7	10~20	4~7
胃肠道				
起始	30~50	1~3	30~50	1~3
维持	10~20	4~7	10~20	4~7
肾脏	20~40	3~5	15~30	3~5
深部裂伤	20~40	5~7	15~30	5~7
手术（大）				
术前	80~100		60~80	
术后	60~80	1~3	40~60	1~3
	40~60	4~6	30~50	4~6
	30~50	7~14	20~40	7~14
手术（小）				
术前	50~80		40~50	
术后	30~80	1~5	10~40	1~5

血友病A首选基因重组FⅧ（rFⅧ）或病毒灭活的血浆源性FⅧ浓缩物[1]–[2]，上述药品不可获得时可临时选用冷沉淀或新鲜冰冻血浆（FFP）等。血友病B首选基因重组凝血因子Ⅸ（rFⅨ）、病毒灭活的血浆源性FⅨ浓缩物或凝血酶原复合物（PCC），上述药品不可获得时可选用FFP等[1]–[2]。

条件允许时可依据个体药代动力学参数调整替代治疗方案[6]。可使用抗纤溶药物作为辅助止血治疗（泌尿系出血慎用）。对于伴抑制物的患者，可使用重组活化人凝血因子Ⅶ（rFⅦa）或PCC等旁路制剂治疗[5],[7]。

近年来，非因子药物越来越多地应用于血友病治疗，当患者使用非因子药物治疗期间发生突破性出血时，应尽快给予凝血因子替代治疗或恰当的旁路制剂止血。对于这类患者，APTT、凝血因子活性等指标可能无法反映患者的真实凝血状况，应结合临床表现和更多的凝血指标进行综合评估和判断并警惕血栓风险[8]。

2. 尽快识别危及生命的大出血或关键部位出血、提高分诊级别：快速识别患者的出血部位，评估生命体征和出血量，对于关键部位出血或急性大出血患者[9]，建议提高急诊分诊级别，尽快进入观察室或抢救室进行诊疗。

大出血定义为至少达到以下一个危险因素[10]：（1）关键部位出血：包括颅内及脊柱内出血，咽喉、气道、心包、腹腔内、腹膜后、消化道出血，以及有致残、致畸风险的关节和肌肉出血。（2）血流动力学不稳定：①心率增快；②收缩压<90 mmHg（1 mmHg＝0.133 kPa）或下降>40 mmHg或体位性血压变化（站立时收缩压下降≥20 mmHg或舒张压下降≥10 mmHg），或平均动脉压<65 mmHg；③器官灌注不足（如尿量<0.5 ml·kg^−1^·h^−1^）。（3）大量出血：HGB下降≥20 g/L或需要输注≥2个单位的浓缩红细胞。

创伤引起的出血有时具有隐匿性及延迟性，其严重程度取决于致伤因素、部位、范围、是否存在创伤性凝血病等[11]，建议对血友病创伤出血患者适当延长急诊留观时间，对于多发伤、复合伤患者，建议转运至有创伤救治及出凝血疾病诊治经验的综合管理中心[2],[5]。对于严重创伤患者，建议在充足替代治疗的情况下尽早实施损伤控制手术（damage control surgery, DCS）[2],[11]。

3. 完善针对性病史采集和选择辅助检查：建议对血友病患者既往诊治情况进行核实。重点询问患者出血发生的时间、部位、症状，是否正在接受预防治疗，基础凝血因子水平和近期抑制物水平检测结果，有无肝、肾及心脏疾病等，是否存在药物、创伤等诱因。重点针对出血部位进行专项查体[12]。

建议尽快进行凝血因子水平、凝血因子抑制物水平、凝血功能等检测。针对不同出血部位完善X线、超声、CT、MRI等影像学检查[13]。部分患者在足量因子替代治疗下，出血仍得不到控制或无法明确病因，可能需要接受穿刺、介入、内镜及外科手术等有创性诊疗。建议按照围术期凝血因子替代方案，在有专业知识和经验、具有血友病多学科诊疗（MDT）模式的医疗机构实施相关手术或有创操作[2]，避免延误诊疗时机。

对于既往血友病诊断证据不足的出血患者，建议进行APTT纠正试验、全套凝血因子活性、狼疮抗凝物、免疫学指标、血小板功能、血管性血友病因子（VWF）抗原和活性、抑制物等检测，与血管性血友病、获得性血友病、其他凝血因子缺乏症等疾病相鉴别[2],[14]。

4. 兼顾出血相关并发症的处置：血友病患者发生出血后常伴多种并发症，如关节、肌肉出血导致的疼痛和功能障碍，中枢神经系统出血导致的发热、癫痫、呼吸循环衰竭，消化道大出血导致的肾前性肾功能不全、肠道菌群易位，创伤性出血伴发的酸中毒、低体温等。处理血友病患者出血的同时，必须重视并发症的识别和处理。

急性疼痛是血友病患者常见并发症，推荐采用数字评分量表（NRS）、视觉模拟量表（VAS）或麦吉尔疼痛问卷（MPQ）评估疼痛程度。及时、足量的凝血因子替代治疗可以有效缓解患者的疼痛。对于关节、肌肉出血的患者，在因子替代治疗的基础上，建议予以患者“PRICE”治疗，即保护（Protect）、休息与制动（Rest）、局部冰敷（Ice）、加压包扎（Compression）和抬高患肢（Elevation）[15]。遵循世界血友病联盟（WFH）推荐的三阶梯治疗原则选择镇痛药物，即首先选用对乙酰氨基酚，若无效可选用COX-2抑制剂（如塞来昔布）或对乙酰氨基酚联合可待因/曲马多，最后可选择强阿片类（如吗啡缓释片）。尽量避免使用阿司匹林以及含有水杨酸的药物，尽量避免肌肉注射[16]。

血友病出血患者在凝血因子替代治疗的前提下，应根据具体疾病的相关诊疗规范，密切监测和评估患者生命体征、容量负荷、内环境、脏器功能及灌注、感染等，按需合理使用血管活性药、抗生素、红细胞输注等，积极维持患者正常体温，必要时实施气管插管、机械通气和肾替代等支持治疗。

5. 重视MDT协作诊疗、实施个体化综合管理：建议根据出血部位及严重程度进行个体化管理，必要时应迅速启动MDT团队协同管理。根据患者出血部位和并发症，MDT团队可由急诊科、血液科、检验科、输血科、骨科、重症医学科等相关科室组成。对于危及生命的急重症出血，必要时可启动远程会诊，获得上级诊疗中心的指导，也可在给予初始治疗病情稳定后通过绿色通道及时转诊[17]。

推荐：

推荐意见1：创伤或者出血发生后，建议尽快（出血后1 h内）开始凝血因子替代治疗，不能因等待检查结果而推迟凝血因子替代治疗。

推荐意见2：尽快识别危及生命的大出血或关键部位出血。

推荐意见3：应关注血友病出血相关并发症，建议在MDT团队协作下，遵循各专科疾病诊疗常规开展诊治。

二、血友病各部位出血的急诊管理

1. 中枢神经系统出血：中枢神经系统出血是血友病患者死亡的主要原因，出血原因包括创伤性出血和自发性出血，其中自发性脑出血占35％～58％[18]。血友病患者创伤性脑出血有时具有延迟性特点，当怀疑脑出血时，即便患者无确诊证据，也建议按照脑出血立即开始凝血因子替代治疗（详见表1、表2），至少观察24～48 h。脑出血后4～6 h是抢救的黄金时间。

头颅CT是诊断早期脑出血的“金标准”[2],[19]，必要时可选择脑动脉CT造影（CTA）、脑磁共振血管造影等检查。对于怀疑脊髓周围出血的患者，可行全脊柱CT或MRI检查以明确。建议使用GCS评分和脑出血评分等进行危险度评估[19]。

除凝血因子替代治疗外，应予以脑出血患者持续生命体征监测，动态评估神经系统功能，按需进行镇静、镇痛、降低颅压等治疗及必要的营养支持[20]。高血压、高血糖与颅内出血预后不良密切相关，因此推荐对血友病颅内出血患者进行强化的血压、血糖管理[21]。建议采用冰毯冰帽等方式进行物理降温，必要时予以规范的亚低温治疗，加强脑保护。对于继发感染的患者，建议积极的抗感染治疗。外科手术可以迅速清除血肿、缓解颅高压、解除机械压迫，对于出血量大，中线移位明显或者发生脑疝的患者，建议在充足凝血因子替代治疗的前提下进行手术治疗[20]。

推荐：

推荐意见4：所有头部外伤和有明显头痛症状患者，即使暂时缺乏实验室或者影像学证据，都建议应按照脑出血立即开始替代治疗。

推荐意见5：血友病中枢神经系统出血是致命部位出血，应尽快开始凝血因子替代治疗。

推荐意见6：推荐在MDT团队协作下针对患者合并症或并发症（高血压、糖尿病、脑疝、感染等）进行诊疗。

推荐意见7：对于出血量大、有手术指征患者应积极创造条件进行手术治疗。

2. 颈部、咽喉部（含口鼻腔）出血：血友病患者颈部、咽喉部、口鼻腔出血多由颈部软组织损伤、黏膜或血管出血、严重扁桃体感染等原因造成，表现为颈部肿胀、呼吸困难、吞咽困难、咽痛、咳嗽、咯血等，严重者可表现为气道梗阻，甚至发生窒息和呼吸衰竭[22]。对于颈、咽喉部出血的患者，建议立即提高凝血因子水平至80％～100％，并维持凝血因子水平直至症状缓解[2]。必要时请耳鼻喉科或口腔科进行局部烧灼、局部喷洒药物（抗纤维蛋白溶解剂、肾上腺素）等治疗，与过敏有关的出血建议同时使用抗组胺药[23]。由于血友病患者咽喉部出血往往继发/伴发严重的扁桃体感染，建议因子替代治疗的同时予以抗生素治疗[2]。治疗过程中，注意维持患者呼吸道通畅，必要时予以气管插管或环甲膜穿刺。

推荐：

推荐意见8：颈咽喉部出血是致命部位出血，应尽快开始凝血因子替代治疗。

推荐意见9：咽喉部感染是出血的高危因素，建议同时抗感染和凝血因子替代治疗直至局部感染控制。

3. 呼吸系统出血：血友病患者可因创伤、气道异物、肺血管疾病或微生物感染诱发呼吸系统出血，偶尔可发生自发性出血。临床上表现为胸痛、呼吸困难、咯血等，严重者出现呼吸衰竭、大咯血窒息、失血性休克等危及生命。推荐通过病原学、免疫学、影像学等在内的辅助检查寻找出血的病因，必要时尚需进行组织活检。纤维支气管镜检查是诊断和治疗呼吸系统出血的重要手段，应在足量凝血因子替代治疗下积极创造纤维支气管镜检查条件[2]。

对于咯血及胸腔出血的患者，建议给予足量凝血因子替代治疗（详见表1、表2），出血量大时可以考虑穿刺引流，同时根据出血原因，予以抗感染、血管栓塞等对因治疗。

推荐：

推荐意见10：重视血友病患者呼吸系统出血的症状和体征，积极在凝血因子替代治疗下寻找出血的病因，并针对病因进行治疗。

4. 腹腔/盆腔出血：血友病患者腹腔/盆腔出血（含腹膜后出血）相对少见，常见病因包括创伤、自发性腹盆腔脏器出血、腹膜后软组织出血、肿瘤破裂出血，部分患者由于围手术期未进行规范、足量的凝血因子替代治疗而发生腹部手术切口出血和创口不愈合。腹腔/盆腔出血临床表现多样，具有延迟性和隐匿性，容易误诊为腹腔感染或阑尾炎、胰腺炎等其他急腹症[2]。需要特别警惕的是腹膜后出血，这是血友病患者腹腔隐性出血的主要部位，出血来源可以是腹膜后脏器组织血管出血，也可以是髂腰肌出血沿腹膜后扩散所致。腹膜后出血症状不典型，常表现为脐周弥漫性疼痛或下腹疼痛，常向股部、背部放射，典型的保护性体位为屈髋屈膝位，极易误诊和漏诊。短期内大量血液流入腹膜后腔隙，有发生神经压迫、失血性休克甚至危及生命的可能。

一旦怀疑腹腔/盆腔腔出血，必须立刻开始足量凝血因子替代治疗（表1、表2）。治疗过程中，推荐动态监测血红蛋白及凝血功能等指标，建议通过腹部超声及CT等检查，明确出血的部位、原因及范围[24]。对于血流动力学不稳定的患者，应积极进行容量复苏，按需输注成分血，必要时应用血管活性药物。对于腹腔/盆腔腔出血，目前主张以内科保守治疗为主，但对于有明确外科手术或介入治疗指征的腹腔/盆腔腔出血，建议MDT讨论和评估必要性后予以实施。

推荐：

推荐意见11：一旦怀疑腹腔/盆腔腔出血，必须立刻开始足量、足疗程的凝血因子替代治疗。

推荐意见12：部分腹腔出血易漏诊（如腹膜后出血）或与其他急腹症混淆，推荐腹部超声及CT检查进行诊断和鉴别。

推荐意见13：血友病腹腔/盆腔出血推荐MDT协作诊疗。

5. 消化道出血：消化道出血的典型临床表现为呕血、黑便、便血等。但在急性出血早期或慢性消化道出血时，患者可仅表现为头晕、乏力、心悸、晕厥等非典型症状，对于症状不典型、存在无法解释的急性贫血患者，应考虑消化道出血的可能[25]。

根据患者生命体征，结合格拉斯哥-布拉奇福德评分（Glasgow-Blatchford score, GBS）进行危险分层，其中存在活动性出血、循环衰竭、呼吸衰竭、意识障碍、发生误吸或GBS>1分中任意一项的患者，应诊断为危险性急性上消化道出血[26]。

对于急性消化道出血的血友病患者应予以积极的凝血因子替代治疗（表1、表2）。同时予以积极的容量复苏，出血未控制时推荐采用限制性液体复苏和允许性低血压复苏策略，即收缩压维持在80～90 mmHg为宜，出血控制后维持至患者基础血压[25]。对于充分液体复苏仍存在低血压的患者，可应用血管活性药物。大量失血患者需适当输注红细胞等血液制品，以保证组织供氧和维持正常凝血功能。

急诊初始处置后应全面评估判断患者消化道出血的病因，最常见的是消化性溃疡，其次为肝硬化门脉曲张破裂出血、消化道肿瘤、应激性溃疡、急慢性上消化道黏膜炎症等。因此在给予凝血因子替代治疗的同时，应进行消化道出血的常规治疗，推荐静脉应用质子泵抑制剂（PPI）和生长抑素治疗，高度怀疑静脉曲张破裂出血的患者，推荐预防性使用抗生素[25]。对于急性非静脉曲张性上消化道出血，建议在出血后24 h内进行内镜检查[24]，药物治疗后血流动力学持续不稳定的患者应进行紧急内镜检查和内镜下止血治疗，有内镜检查禁忌、药物及内镜治疗失败仍有活动性出血或腹部CTA提示出血的患者，可急诊介入检查治疗[27]。检查治疗前后应参考围手术期凝血因子替代治疗方案输注凝血因子（表1、表2）。

推荐：

推荐意见14：消化道出血是血友病患者致命部位出血，应尽快开始凝血因子替代治疗，同时针对消化道出血病因进行治疗，推荐使用抑酸药及生长抑素。

推荐意见15：建议在MDT协作下诊疗，除凝血因子替代治疗外，应重视分层救治、危险评估、容量复苏等。

推荐意见16：积极为血友病消化道出血患者创造条件进行消化内镜等有创诊疗。

6. 关节出血：关节是血友病患者最常见的出血部位，占所有出血的70％～80％，多见于重型及中间型患者，也可见于轻型患者外伤、剧烈活动后[2],[5]。关节出血是患者致残的主要原因。

血友病关节出血常用检查方法是超声[28]。因价格低廉、检查便捷、重复性好等优点，超声检查更适合血友病关节出血的急诊诊断和评估。若关节出血的同时出现局部红肿热痛或伴高热，应警惕发生化脓性关节炎可能，应进行感染学指标及微生物学检查（常见病原菌为革兰阳性球菌）。

急诊科是处置血友病关节出血的重要场所，早期开始凝血因子替代治疗可有效止血，减轻关节疼痛和关节损伤，降低远期致畸和致残风险。因此，一旦怀疑关节出血就应开始凝血因子替代治疗[28]（表1、表2）。关节出血患者还应接受“PRICE”治疗，伴有剧烈疼痛者应给予有效的镇痛治疗（详见管理原则部分）。

一般不推荐关节穿刺术，仅在充分凝血因子替代治疗下关节出血的症状不缓解或者持续恶化时排除抑制物、合并感染等其他原因后，考虑减压能改善症状方可谨慎进行。关节出血控制、疼痛缓解后，建议尽快进行个体化的康复与物理治疗[2]。

推荐：

推荐意见17：关节出血是血友病最常见的出血类型、是患者致残的主要原因，应积极进行凝血因子替代治疗，并对治疗效果和关节功能进行评估。

推荐意见18：重视关节出血相关并发症（疼痛、感染等）的管理。

推荐意见19：重视关节出血后的康复与物理治疗。

7. 肌肉及软组织出血：血友病患者肌肉及软组织出血的发生率仅次于关节出血，典型表现为疼痛、肿胀、肌肉保护性痉挛、相连关节屈曲及活动受限等，其严重程度与出血部位密切相关。皮肤软组织深部撕裂伤或髂腰肌等大肌群的出血，由于出血常沿着筋膜面扩散，出血范围广，可引起大量失血甚至休克，危及患者生命；腓肠肌、前臂肌群的出血可引起肌肉痉挛、骨筋膜室综合征等并发症，导致神经和肌肉功能损伤，是血友病患者致残的重要原因之一[2]。

血友病患者皮肤软组织的浅表撕裂伤一般不会引起严重后果，仅需要进行清创、加压包扎、冰敷等常规处理。而深部软组织或重要肌肉的出血，应立即接受凝血因子替代治疗，并维持至出血症状体征消失[1],[2]（替代治疗方案见表1、表2）。治疗过程中，应密切监测是否发生神经血管并发症。此外，血友病肌肉出血患者也应该接受“PRICE”治疗和理疗康复治疗。深部组织撕裂伤或髂腰肌出血，需动态评估出血量及血肿范围，必要时在充足凝血因子替代的前提下，经MDT会诊讨论有创治疗（切开引流、减压、外科止血等）的获益和风险。

推荐：

推荐意见20：应尽快识别有致死、致残风险的肌肉出血（髂腰肌出血、前臂肌群出血、腓肠肌出血等），尽早开始凝血因子替代治疗。

推荐意见21：重视肌肉出血后物理及康复治疗。

8. 泌尿系出血：血友病肾/泌尿系出血危险程度较低，临床上主要表现为无症状血尿，也可出现腰痛、尿路刺激症状。对于无症状血尿患者，不必过度凝血因子替代治疗，建议卧床及大量饮水（每天2 L/m^2^体表面积）。症状性血尿、出血量较大或伴有血红蛋白下降的泌尿系出血，应进行凝血因子替代治疗，治疗过程中避免使用抗纤溶药物或其他止血药物，以免形成血凝块，发生阻塞性肾功能衰竭。对反复发生泌尿系出血的患者，建议进行包括泌尿系超声或CT等检查，明确有无泌尿系结石、肿瘤等病因。

推荐：

推荐意见22：血友病泌尿系出血患者进行凝血因子替代治疗时，避免同时使用抗纤溶药，防止发生阻塞性肾功能衰竭。

推荐意见23：反复发生泌尿系出血的患者应重视病因筛查。

## References

[1] 中华医学会血液学分会血栓与止血学组, 中国血友病协作组 (2020). 血友病治疗中国指南(2020年版)[J]. 中华血液学杂志.

[2] 杨 仁池 (2021). 中国血友病管理指南2021版[M].

[3] Ndai AM, Allen BR, Wynn TT (2024). Rapid recognition and optimal management of hemophilia in the emergency department: A quality improvement project[J]. J Am Coll Emerg Physicians Open.

[4] Lee A (2019). Emergency management of patients with bleeding disorders: Practical points for the emergency physician[J]. Transfus Apher Sci.

[5] Srivastava A, Santagostino E, Dougall A (2020). WFH Guidelines for the Management of Hemophilia, 3rd edition[J]. Haemophilia.

[6] 中国血友病协作组 (2021). 药物代谢动力学指导血友病A治疗的中国专家共识[J]. 中国临床研究.

[7] 中华医学会血液学分会血栓与止血学组, 中国血友病协作组 (2023). 血友病合并抑制物诊断与治疗中国指南(2023年版)[J]. 中华血液学杂志.

[8] 侯 鹏霄, 杨 仁池 (2023). 血友病替代治疗研究新进展[J]. 中华血液学杂志.

[9] Franco L, Becattini C, Beyer-Westendorf J (2020). Definition of major bleeding: Prognostic classification[J]. J Thromb Haemost.

[10] Tomaselli GF, Mahaffey KW, Cuker A (2020). 2020 ACC Expert Consensus Decision Pathway on Management of Bleeding in Patients on Oral Anticoagulants: A Report of the American College of Cardiology Solution Set Oversight Committee[J]. J Am Coll Cardiol.

[11] 中国人民解放军急救医学专业委员会, 中国医师协会急诊医师分会, 北京急诊医学学会 (2023). 创伤失血性休克中国急诊专家共识(2023)[J]. 临床急诊杂志.

[12] Alblaihed L, Dubbs SB, Koyfman A (2022). High risk and low prevalence diseases: Hemophilia emergencies[J]. Am J Emerg Med.

[13] Mansouritorghabeh H (2015). Clinical and laboratory approaches to hemophilia A[J]. Iran J Med Sci.

[14] Tiede A, Collins P, Knoebl P (2020). International recommendations on the diagnosis and treatment of acquired hemophilia A[J]. Haematologica.

[15] Zhang L, Zhang P, Chen W (2023). Treatment regimens, patient reported outcomes and health-related quality of life in children with moderate and severe hemophilia A in China: using real-world data[J]. Orphanet J Rare Dis.

[16] Auerswald G, Dolan G, Duffy A (2016). Pain and pain management in haemophilia[J]. Blood Coagul Fibrinolysis.

[17] St-Louis J, Chowdary P, Dolan G (2022). Multidisciplinary Team Care of Patients with Hemophilic Arthropathy: A Qualitative Assessment of Contemporary Practice in the UK and Canada: Canada/UK: MDT Practices for Hemophilia[J]. Clin Appl Thromb Hemost.

[18] Zwagemaker AF, Gouw SC, Jansen JS (2021). Incidence and mortality rates of intracranial hemorrhage in hemophilia: a systematic review and meta-analysis[J]. Blood.

[19] Gil-Garcia CA, Flores-Alvarez E, Cebrian-Garcia R (2022). Essential Topics About the Imaging Diagnosis and Treatment of Hemorrhagic Stroke: A Comprehensive Review of the 2022 AHA Guidelines[J]. Curr Probl Cardiol.

[20] 中华医学会神经病学分会, 中华医学会神经病学分会脑血管病学组 (2019). 中国脑出血诊治指南(2019)[J]. 中华神经科杂志.

[21] Hemphill JR, Greenberg SM, Anderson CS (2015). Guidelines for the Management of Spontaneous Intracerebral Hemorrhage: A Guideline for Healthcare Professionals From the American Heart Association/American Stroke Association[J]. Stroke.

[22] Tebo C, Gibson C, Mazer-Amirshahi M (2020). Hemophilia and von Willebrand disease: a review of emergency department management[J]. J Emerg Med.

[23] 中华耳鼻咽喉头颈外科杂志编辑委员会鼻科组, 中华医学会耳鼻咽喉头颈外科学分会鼻科学组 (2022). 中国变应性鼻炎诊断和治疗指南(2022年, 修订版)[J]. 中华耳鼻咽喉头颈外科杂志.

[24] Barkun AN, Almadi M, Kuipers EJ (2019). Management of Nonvariceal Upper Gastrointestinal Bleeding: Guideline Recommendations from the International Consensus Group[J]. Ann Intern Med.

[25] 中国医师协会急诊医师分会, 中华医学会急诊医学分会, 全军急救医学专业委员会 (2021). 急性上消化道出血急诊诊治流程专家共识[J]. 中国急救医学.

[26] Laursen SB, Dalton HR, Murray IA (2015). Performance of new thresholds of the Glasgow Blatchford score in managing patients with upper gastrointestinal bleeding[J]. Clin Gastroenterol Hepatol.

[27] Wells ML, Hansel SL, Bruining DH (2018). CT for Evaluation of Acute Gastrointestinal Bleeding[J]. Radiographics.

[28] 中华医学会骨科学分会关节外科学组, 中国血友病协作组 (2023). 中国血友病骨科手术围手术期管理指南[J]. 中华骨科杂志.

